# Extending Theory of Planned Behavior to Understand Service-Oriented Organizational Citizen Behavior

**DOI:** 10.3389/fpsyg.2022.839688

**Published:** 2022-04-08

**Authors:** Kuang-Chung Tsai, Tung-Hsiang Chou, Santhaya Kittikowit, Tanaporn Hongsuchon, Yu-Chun Lin, Shih-Chih Chen

**Affiliations:** ^1^Department of Safety, Health and Environmental Engineering, National Kaohsiung University of Science and Technology, Kaohsiung, Taiwan; ^2^Department of Information Management, National Kaohsiung University of Science and Technology, Kaohsiung, Taiwan; ^3^Chulalongkorn Business School, Chulalongkorn University, Bangkok, Thailand

**Keywords:** theory of planned behavior, organizational justice, job attitude, structural equation modeling, service-oriented organizational citizenship behavior

## Abstract

The financial crisis of 2007–2008 and the COVID-19 pandemic have caused many enterprises to suffer great losses. Thus, companies have to take measures such as pays cut, furloughs, or layoffs, which caused dissatisfaction among employees and triggered labor disputes. Therefore, this study explores the service-oriented organizational citizenship behavior based on the decomposed theory of planned behavior in order to understand the behavioral intentions of employees through their mental states, job attitudes, subjective norms, and perceived behavioral control. This study conducted questionnaire surveys for employees in different industries, collected 281 valid questionnaires, and applied Structural Equation Model for the analysis. The results show: (1) employees believe organizational justice in the organization is important, and when they feel treated fairly, their job attitudes and beliefs are enhanced. (2) Employees’ job attitudes and beliefs support service-oriented organizational citizenship behavior, in other words, they have positive job attitudes and beliefs and will actively provide better service to customers. (3) When employees are treated reasonably and fairly by the organization and have positive job attitudes (job satisfaction and organizational commitment) and perceived behavior control, their spontaneous service-oriented organizational citizenship behavior is stimulated, thus increasing organizational development.

## Introduction

As the COVID-19 pandemic spread globally in 2020, many countries and cities adopted lockdowns or similar measures to prevent the spread of this epidemic. Many people have been forced to stay at home, leading to great changes in their lives, such as working at home, even leading to fear of leaving home. When business slumps, an enterprise must cut spending (e.g., reducing working hours or wages), or even shut down. The magnitude of the unpaid leave due to COVID-19 is much larger than the financial crisis of 2007–2008. In addition to being afraid of catching the disease, employees worry about reduced income, unemployment, and unfair treatment by the organization. These psychological factors of fear or panic, as well as attitude towards employers may affect their performance and behavioral intentions, and employees’ performance will affect the organization’s performance (Kang et al., 2021; Wong et al., 2021). The most direct example is frontline service staff. Because they have most often contact with customers, they communicate information about the organization to customers. Customers may feel that the behavior of service staff represents the company, so their spontaneous and positive performance can benefit the organization. Therefore, this study focuses on service-oriented organizational citizenship behavior.

Many studies have applied the theory of planned behavior (TPB) to explore organizational citizenship behavior (OCB; e.g., Jung and Yoon, 2015; Radaelli et al., 2015; Aguiar-Quintana et al., 2020). These demonstrated that behavioral beliefs such as attitudes, subjective norms, and perceived behavior control influence OCB. Taylor and Todd (1995) proposed the decomposed theory of planned behavior (DTPB), decomposing some factors that affect behavior in the original theory, so it is more flexible than TPB. As mentioned above, the psychology of employees is an important factor affecting their action intentions. Previous studies have pointed out a correlation between organizational justice and civic behavior (e.g., Saifi and Shahzad, 2017). Employees’ perception of organizational justice affects their job attitude and behavior. Thus, this research uses disaggregated planning behavior theory to explore the impact of organizational justice on service-oriented OCB and to understand how employees’ perceptions of the organization affect their behavioral intentions. The next section describes DTPB and related research. In the third section, the research framework is proposed, conducting model validation is explained in the fourth section, and conclusions are presented in the final section.

## Literature Review

This study applies social psychology to explore job attitude and behavior of employees. In order to more effectively and correctly understand relationships that affect organization members, this study uses DTPB to explore the behavior of employees and the impact of organizational justice on employee psychology. This section reviews relevant literature and empirical research.

### Decomposed Theory of Planned Behavior

In decomposed theory of planned behavior (DTPB) the belief structure underlying planning behavior theory is decomposed, as an evolution of TPB. DTPB was proposed by Taylor and Todd (1995) based on TPB (Ajzen, 1985) and the technological acceptance model (TAM; Davis, 1989; Davis et al., 1989), adding referent groups from innovation diffusion theory (Rogers, 1983). It also includes concepts of self-efficacy and facilitating condition; and it decomposes beliefs such as attitude, subjective norms, and perceived behavior control into multi-dimensional belief variables.

Bagozzi (1981, 1982, 1983) found that multidimensional belief structures are more suitable than unidimensional constructs to describe the effects of behavioral attitudes. Some studies have also argued that a multi-dimensional belief structure is indeed more appropriate to explain “behavioral attitudes” (Bagozzi, 1981; Shimp and Kavas, 1984). In addition, the basis of TPB and TAM are both developed from the theory of reasoned action (TRA; Fishbein and Ajzen, 1975).

As mentioned above, the model of DTPB is used to decompose the TPB, whose main factors include attitude, subjective norm, perceived behavioral control, and behavioral intention. “Attitude” refers to an individual’s perception of good or bad, positive or negative, when engaging in a behavior. People’s attitudes toward a behavior are influenced by their “Behavioral Beliefs” and “Outcome Evaluation” (Fishbein and Ajzen, 1975). “Subjective norm” refers to the social or reference group pressure that an individual perceives when engaging in a particular behavior (Ajzen and Fishbein, 1975, 1980; Lee and Green, 1991). “Perceived behavior control” refers to an individual’s ability to control opportunities and resources when engaging in a behavior (Ajzen, 1989). “Behavior intention” refers to the intensity of a person’s intention to engage in a behavior and is usually used to predict or explain actual behavior (Fishbein and Ajzen, 1975). Attitudes, subjective norms and perceived behavior control influence behavior intentions.

Taylor and Todd (1995) argued that the advantages of using DTPB are that (1) the relationship between predispositions and dimension of belief is clearer and (2) it is easier to identify specific contributing factor s because of the consistency between predispositions and dimensions of belief (Bagozzi, 1981; Shimp and Kavas, 1984). Thus, it is easier to understand the individual predispositions of different belief dimensions.

TPB has been an important theory for exploring human behavioral intentions and as a basis for discussing a wide range of issues such as employee behavior (e.g., Islam et al., 2020; Jin et al., 2021) and purchase intention (e.g., Ruangkanjanases et al., 2020). Moreover, as mentioned above, the multi-dimensional framework is more suitable for explaining behavioral intentions. Scholars have subsequently added other factors to TPB (e.g., Duan, 2022) or combined TPB with other theories (e.g., Luo et al., 2021), extending research on TPB. Hence, this study adopts DTPB as the theoretical basis for discussing factors that influence services-oriented organizational citizenship behavior.

### Job Attitude

In DTPB, an important factor affecting behavioral intention is attitude (Taylor and Todd, 1995). Social psychologists consider that attitudes can be divided into affective and cognitive. The former reflects the individual’s feelings about a particular object, such as liking or disliking the work they are engaged in; the latter is whether the particular object can reflect personal thoughts and beliefs, such as whether the work performed can satisfy one’s own expectations. Thus, a person’s “attitude” toward something can be used to predict to what extent the person will engage in a behavior (Fishbein and Ajzen, 1975).

Attitude means an employee’s positive or negative evaluation of people, events, and things that influences his or her behavior (Robbins, 1996). Robbins (1993) argues that employees’ attitudes are critical in an organization because they affect performance. Steers and Black (1994) state that job attitudes can be categorized into three concepts: job satisfaction, organizational commitment, and work participation. Martin and Bennett (1996) find that job satisfaction and organizational commitment are very important variables in the studies of organizational behavior. In many studies on behavior and job attitudes, job satisfaction and organizational commitment are central topics (Currivan, 1999; Dirks and Ferrin, 2002; Harrison et al., 2006). Studies have also found that job attitudes composed of organizational commitment and job satisfaction can be used to anticipate employee behavior (e.g., Moon, 2000; Harrison et al., 2006; Lu et al., 2010). Thus, according to the viewpoints of most scholars on the influencing factors of job attitude, this study divides it into the dimensions of job satisfaction and organizational commitment.

#### Job Satisfaction

Since Hoppock (1935) put forward the concept of job satisfaction, scholars have developed different definitions of job satisfaction. Most interpretations of this aspect revolve around work-oriented or overall emotional responses (Kreitner and Kinichi, 1995). In addition, some scholars consider that job satisfaction refers to workers’ subjective attitudes toward their workplace (Dunham and Herman, 1975; Jayaratne, 1993; Murphy et al., 2002; Barling et al., 2003). Other studies have proposed aspects to measure job satisfaction, which can be roughly divided into external satisfaction (e.g., workplace, salary and compensation, employee bonus, welfare system, peer relationship, leadership style) and internal satisfaction (e.g., recognition, achievement, autonomy, learning, promotion, and honor from work; Weiss et al., 1967). This classification has been empirically verified (Arvey et al., 1989).

#### Organization Commitment

Organizational commitment was first conceptualized by Whyte (1956) and has become a widely discussed topic among management scholars. Whyte believed that members of an organization not only work for the organization, but also are part of the organization. Therefore, they identify with the values and targets of the organization, commit themselves to their work, and demonstrate loyalty (Sheldon, 1971; Buchanan, 1974). Organizational commitment is valued since high organizational commitment benefits organizational development by supporting the contributions and loyalty of employees to the organization. Porter et al. (1974) suggested that organizational commitment could anticipate turnover. Morris and Sherman (1981) thought it could also predict employee performance. Ferris and Aranya (1983) argued that it could also be used as an indicator of organizational performance.

Due to different research purposes, scholars have various definitions of organizational commitment. Steers (1977) pointed out that social psychologists and organizational behavior researchers have different perspectives on organizational commitment. One claim is “behavior commitment,” and the other is “attitude commitment.” Behavioral commitment is a belief that an organization’s members are influenced by their past behavior and continue to be committed to their work (Salancik, 1977); attitudinal commitment is an indication that an organization’s members display certain attitudes toward the organization. Other scholars have separated organizational commitment into effort commitment, value commitment, and retention commitment from the perspective of emotional attachment (Porter et al., 1974). Effort commitment refers to members’ willingness to work hard for the benefit of the organization; value commitment is the belief that members accept the targets and values of the organization; retention commitment is the strong desire of members to maintain their job opportunities (Mowday et al., 1982). Scholars also discuss organizational commitment from the viewpoints of behavior, attitude, values, exchange, and ethics. Morrow (1983) pointed out that there are at least 25 concepts and measurement methods related to organizational commitment. However, organizational commitment is commonly defined by attitude (Schwepker Jr., 2001; Spector et al., 2002; Jaramillo et al., 2005).

This study uses organizational commitment defined by the emotional attachment perspective (Porter et al., 1974). This concept emphasizes employees’ recognition of organizational goals and values, more effort and high work commitment, loyalty, and willingness to remain in the organization. Organizational commitment is divided into three factors: effort commitment, value commitment, and retention commitment.

### Organizational Justice

From the perspective of human resources, organizations should consider the opinions of employees that affect their attitudes and behaviors toward work. Previous studies have used “organizational justice” to assess how much importance employees attach to the organization (e.g., Colquitt et al., 2001). Scholl et al. (1987) suggested that organizational justice means the subjective perceptions of employees regarding the justice of the organization in allocating resources or determining various rewards and sanctions. Organ (1988) believed that organizational justice is closely related to individual behavior, and whether managers are fair or not affects employees’ behavior. Organizational justice is one of the main factors affecting employee behavior, and it is also a management issue that managers must consider. In the study of organizational behavior, it is a topic that has been widely discussed. Some scholars believe that “justice” is a very important characteristics of organizations and also an important factor affecting employee behavior (Dittrich and Carrell, 1979; Niehoff and Moorman, 1993; Masterson et al., 2000). Schermerhorn (1996) argued that organizational justice is an indicator that can be used to explain whether employees are satisfied with their work status. If the reward is proportional to the effort, it has a positive effect; otherwise, it causes dissatisfaction and reduces performance. Erdogan et al. (2006) also emphasized that employees’ perceptions of justice are strictly related to leaders’ behaviors and attitudes. In short, organizational justice explores subjective perceptions of employees regarding the fairness of resource allocation and managers’ decisions on rewards and punishments. When members of an organization interact, employees often follow this principle to evaluate whether their input and output are proportional. When employees perceive the organization treats them fairly, they adjust their behaviors and attitudes, supporting organizational performance.

Reviewing the theoretical development of organizational justice, previous scholars focused on the discussion of “distributive justice” (Homans, 1961; Adams, 1965). Homans (1961) defined distributive justice as fairness in the distribution of interpersonal rewards and costs and believed that employees feel they are being treated fairly if their contribution is expected to be proportional to the allocation of organizational resources; otherwise Adams (1965) also focused on the concept of distributive justice. However, organizational justice does not have only a single dimension, and its dimensions vary according to the situations and theoretical interpreations. For example, Thibaut and Walker (1975) posited that a court’s judgment process affects people’s recognition of the judgment result. This, then, leads to the appearance of procedural fairness, which results in the dual aspects of distributive and procedural justice. Bies and Moag (1986) argued that previous studies had ignored the importance of interpersonal interactions and therefore proposed three constructs of distributive, procedural, and interactional justice. Since then, a more complete structure of organizational justice has been developed, generally known as the traditional three constructs. Subsequently, scholars have proposed four or six dimensions of organizational justice (e.g., Alexander and Ruderman, 1987; Colquitt et al., 2001), but the traditional three-dimensional approach is still used in most studies. This research also adopts distributive, procedural, and interactional justice to discuss.

### Service-Oriented Organizational Citizenship Behavior

Service-oriented organizational citizenship behaviors (Service-Oriented OCB) is an extension of OCB (Bettencourt et al., 2001) that refers to the attitudes and behaviors of front-line service staff who are enthusiastic and responsible in providing services to match the needs of customers. In an early study of OCB, Barnard (1938) argued that the success of an organization depends on the cooperation of its members. This kind of cooperative behavior requires the operation of some informal organizations to be effective, so the positive behavior required by non-organizations is also an important theoretical basis for OCB.

Service-oriented means that service staff provide high-quality services to meet customer needs through enthusiasm, courtesy, and sincerity. It also combines staff capability, willingness to learn, motivation and attitude (Hogan et al., 1984). In the service industry, customers’ perception of service quality comes through interactions with frontline service staff. Therefore, in addition to valuing employees’ OCB, service-oriented OCB is even more important for this industry (Borman and Motowidlo, 1993). Bettencourt et al. (2001) reviewed research on service-oriented OCB and suggested that the evaluation of service quality and performance should include service behavior from a non-technical perspective. Service-oriented OCB is a new type of service that refers to attitudes of enthusiasm, responsibility, and courtesy displayed by front-line service personnel in providing services. This kind of OCB to meet the needs of customers is customer-oriented behavior, so non-technical service behavior shows that service-oriented OCB is an extension of OCB. Service quality is the subjective perception of the service delivery process and the actual result of the service received by the customer, which is a key factor of competitive advantage (Liebermann and Hoffmann, 2008).

Service-oriented OCB has the three important connotations of loyalty, service delivery, and participation (Van Dyne et al., 1995; Bettencourt et al., 2001). Loyalty refers to employees who provide services/goods and show support for the organization, which may enhance or detract from the organization’s image (Schneider and Bowen, 1985; Bettencourt et al., 2001). Service delivery means that in the process of service delivery, front-line service personnel demonstrate courteous, reliable, dutiful, dedicated, and trustworthy behaviors (Parasuraman et al., 1988; George, 1991). Therefore, service delivery is an important factor that affects customer satisfaction, overall perception of service quality, and even customer loyalty (Bettencourt et al., 2001). Finally, participation means that when employees contact customers, they are the main source of information for customers and are the customer’s first and main impression of the organization. By participating in services, employees can provide management suggestions for improving service quality to enhance customer purchase intention and satisfaction (Schneider and Bowen, 1985; Parasuraman et al., 1988; George, 1991; Heskett et al., 1994; Bettencourt et al., 2001). Therefore, this study takes loyalty, service delivery and participation as factors of service-oriented OCB.

In summary, the operational definitions of the variables are organized as shown in Table 1.

**Table 1:** Variable definition.

Dimensions	Variables	Description	References
Organizational justice	Distributive justice	That is, employees’ perceptions of workload, resource allocation, responsibility and fairness of reward distribution.	Greenberg, 1990; Niehoff and Moorman, 1993
	Procedural justice	That is, employees’ awareness of whether the needs of employees are valued in the process of organizational decision-making.	
	Interactional justice	That is the interaction of interpersonal relationships. It is the perception of whether employees are treated fairly by the organization members and the workplace.	
Job satisfaction		It refers to the satisfaction that workers get from the work content, workplace, organizational system and organizational culture.	Weiss et al., 1967
Organization commitment	Effort commitment	This refers to the employees’ beliefs about the organization’s values and goals.	Porter et al., 1974; Mowday et al., 1982
	Value commitment	Refers to the willingness of employees to work hard to pursue organizational benefits.	
	Retention commitment	It refers to the strong desire of employees to keep their jobs.	
Subjective norm		The external social or reference group pressure that employees feel when they want to adopt a certain behavior.	Ajzen and Fishbein, 1975, 1980; Lee and Green, 1991
Perceived behavior control	Belief	The belief that employees believe they can achieve their organizational targets with the intention of achieving them.	Bandura, 1977; Taylor and Todd, 1995
	Capability	The extent to which employees believe they have sufficient capability to achieve the organization’s goals.	
Service-oriented organizational citizenship behavior	Loyalty	In addition to providing good service quality, employees’ recognition of maintaining and improving corporate image.	Van Dyne et al., 1995; Bettencourt et al., 2001; Liebermann and Hoffmann, 2008
	Service delivery	The degree of satisfaction of employees with their performance when providing services to customers.	
	Participate	Employees are involved in improving the level of customer satisfaction when providing services.	

## Research Model and Data Collection

Based on the literature discussion, this study proposes research hypotheses and constructs research models. The process of collecting measurement data and analysis tools are explained below.

### Hypothesis and Framework

Lawler and Hall (1970) stated that when a person expects to receive fair treatment for their performance, they work hard and further develop job satisfaction. Folger and Konovsky (1989) found that both procedural and distributive justice were significantly correlated with organizational commitment, and that procedural justice predicts organizational commitment more than distribution fairness does. Moorman (1991) found that organizational justice can significantly affect job satisfaction and furthermore: salary satisfaction has a significant positive relationship with distribution justice, promotion, and organizational commitment; that procedural justice is positively related to managerial satisfaction, organizational commitment, and work engagement; and that procedural justice and distributive justice is useful in predicting factors such as employee job satisfaction, organizational commitment, and work commitment. Zahednezhad et al. (2021) indicated that organization justice (including distributive justice and interactional justice) positively and significantly affected nurses’ job satisfaction. Additionally, many empirical studies have found that organizational justice significantly directly affects organizational commitment (Martin and Bennett, 1996; Rahman et al., 2016; Aguiar-Quintana et al., 2020). Thus, this study predicts that employees’ perception of organizational justice should influence job attitudes, leading to the following hypothesis.

*Hypothesis 1*: Employees’ perception of organizational justice has a significant impact on job satisfaction.

*Hypothesis 2*: Employees’ perception of organizational justice has a significant impact on organizational commitment.

According to Adams (1965) equity theory, employees must perform well within organizational norms in order to receive more compensation. In an organizational environment, the three most important reference groups are colleagues, supervisors, and subordinates. For example, perceived organizational support means that employees recognize what behavior the organization wants them to perform or what employees they become under the organizational norms (Eisenberger et al., 1986). Ibragimova (2006) applied TRA to explore whether organizational justice affects the sharing of corporate knowledge and found that organizational justice has a positive relationship with subjective norms. Consequently, this study predicts a relationship between employees’ perceptions of organizational justice and subjective norms and proposes the following hypothesis.

*Hypothesis 3*: Employees’ perception of organizational justice has a significant impact on subjective norm.

Edwards (1991) researched on “personal-work fit” and believed that job supply includes salary, benefits, training, decision-making participation, and job richness, etc., and it is the source of organizational justice. For example, pay and benefits determine distributional justice and decisional involvement determines procedural justice. In terms of beneficial environmental factors, from the perspective of personal cognition, people’s perception is affected by the fair distribution of organizational resources and rewards. The fairer the distribution of external environmental resources and remuneration, there is no doubt that people think that they are treated fairly and the easier it is to accomplish organizational tasks. Hence, this study infers that employees’ perception of organizational justice should have a significant influence on perceptual behavior control, and then put forward the following hypothesis.

*Hypothesis 4*: Employees’ perception of organizational justice has a significant impact on perceived behavior control.

Bettencourt and Brown (1997) found that job satisfaction and organizational justice positively influenced employee service-oriented citizenship behavior, resulting in higher customer satisfaction with service delivery. Bettencourt et al. (2001) also found that job satisfaction can effectively predict some service-oriented OCB. Other studies have found a relation between job satisfaction and OCB (Jung and Yoon, 2015; Torlak et al., 2021). Another study found that organizational justice (distributive and interactional justice) was significantly and positively related to nurses’ job satisfaction; and job satisfaction was significantly and negatively related to turnover intentions (Zahednezhad et al., 2021). There is also support for the finding that job satisfaction is a mediating variable between organizational justice and OCB (Saifi and Shahzad, 2017). Furthermore, organizational commitment has a significant positive relation with employee behavior (Su et al., 2019). Aguiar-Quintana et al. (2020) found that continuous organizational commitment significantly positively affects interpersonal relationships in OCB. Hasyim and Palupiningdyah (2021) also argue that organizational justice positively affects organizational commitment and further affects OCB. Therefore, this study concludes that job attitudes should also have a significant influence on service-oriented OCB, leading to the following hypothesis.

*Hypothesis 5*: Employees’ job satisfaction has a significant impact on service-oriented OCB.

*Hypothesis 6*: The organizational commitment of employees has a significant impact on the intention of service-oriented OCB.

According to Taylor and Todd (1995) definition of subjective norms, employees’ normative beliefs and motivations to comply are influenced by their peers, supervisors and subordinates. Relationships between peers are the interaction of interpersonal relationships, whereas in the relationship between supervisors and subordinates, subordinates usually abide by the rules of superiors in order to maintain a smooth workplace relationship. Ajzen and Fishbein (1980) pointed out that behavior is sometimes more affected by social environmental pressures than personal attitudes. For example, a supervisor wishes the employees to use a new operating system and also agrees with its functions. At this time, employees have a strong motivation to comply with the supervisor’s expectations, so positive subjective norms are created. Liker and Sindi (1997) explored when to influence employees’ willingness to continue to use expert systems to assist their work, confirming that subjective norms have a positive relationship with behavioral intentions. Bock and Kim (2002) found that subjective norms affect behavioral intentions. Torlak et al. (2021) studied the planned behaviors of nurses and found that subjective norms significantly positively affected burnout and then further significantly negatively affected OCB. Hence, this study concludes that the subjective norms of employees have a significant impact on service-oriented OCB, and puts forward the following hypotheses.

*Hypothesis 7*: The subjective norms of employees have a significant impact on service-oriented OCB.

Internal factors refer to self-efficacy, such as information, skills, abilities, forgetting, emotions, and coerciveness; external factors refer to opportunities to help perform a certain behavior, which are the favorable environment (Ajzen, 1985; Taylor and Todd, 1995). Self-efficacy is considered from the social learning/cognitive theory proposed by Bandura (1977), and is regarded as an element of a special situation related to capability and is a dynamic concept. Self-efficacy can also be said to be the capability and belief that people have when they complete a certain behavior. In other words, self-efficacy is individuals’ belief in their ability to succeed and a judgment on their ability to complete. Employees in an organization have continuous interaction with the environment (e.g., rewards or punishments) to carry out the process of self-regulation, so people’s behaviors differ according to the situation. In short, when a person is engaged in a behavior, he or she can only perform actual behaviors when he or she is sure that he or she can effectively anticipate them. However, there are differences in previous findings regarding the relation between perceived behavioral control and behavioral intentions. Some studies have shown a significant relationship between the two (e.g., Torlak et al., 2021), but others have found no relationship (e.g., Chemseddine and Kamel, 2021). This study considers that frontline employees’ perceived behavior control has a significant impact on service-oriented OCB, so the following hypothesis is proposed.

*Hypothesis 8*: Employees’ perceived behavior control has a significant impact on service-oriented OCB.

**Figure 1:**
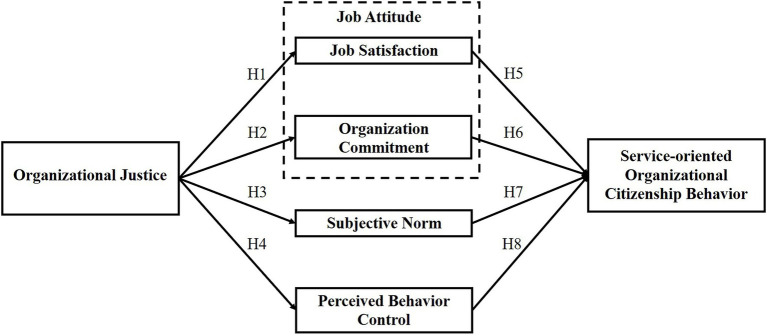
Research model.

Based on the research purpose and literature review, this study argues that organizational justice impacts job attitudes (job satisfaction and organizational commitment), subjective norms, and perceived behavior control, which in turn impact service-oriented citizenship behavior (OCB). Thus, the conceptual framework of this study is constructed, as shown in Figure 1.

### Materials

This study focuses on student workers and general workers because they are the main laborers in society. On-the-job training students use their free time to continue their studies, hoping to enhance their competitiveness with better development in the workplace. The sample of this study includes various fields and industries. The survey was conducted by distributing paper-based questionnaires. The questionnaires were anonymous and distributed by convenience sampling. A total of 315 questionnaires were collected, and invalid questionnaires were deleted. There were 281 valid questionnaires (89.21%).

### Research Method

This study uses SPSS and SmartPLS and selects the appropriate data analysis method based on the research purpose and the measurement level of the variables. To explore the effects between the dimensions and to validate the fit of the research model, a Structural Equation Model (SEM) was used for data analysis to explore the relationship between potential variables and to test the proposed hypothesis. Since a single-stage SEM estimates both the measurement model and the structural model, if the model is not properly matched, it is difficult to judge whether the model error comes from the structural model or the measurement model, or both. For this problem, Anderson and Gerbing (1988) and Williams and Hazer (1986) suggested a two-stage SEM validation procedure, in which the fit of the measurement model is examined first, and then the structural model is examined when the fit of the measurement model reaches an acceptable standard. To use SEM as a measurement method, a theoretical model is proposed and the hypothesis of the relationship between potential variables is described before performing the two-stage SEM validation.

## Research Results

Following Anderson and Gerbing (1988), this study conducts a two-stage SEM analysis. Partial Least Squares Structural Equation Modeling (PLS-SEM) was applied to measure the model and analyze data. SPSS and SmartPLS were used for statistical analysis. The results are described in the following sections, including descriptive statistics, reliability and validity, and path analysis of the model.

### Descriptive Statistics

Before the final questionnaire was distributed, this study first had experts check quality of the questionnaire and then conducted the investigation. A total of 315 questionnaires were collected, 281 questionnaires are valid (89.21%). Descriptive statistics of the valid samples are shown in Table 2. There were more females (66.55%) than males (33.45%); more single (84.70%) than married (15.30%); respondents aged 21–25 were the largest group (35.59%), followed by those 26–30 (17.08%).; the highest percentage education level is college/university (96.09%); those with work experience of 1–5 years were the largest group (48.40%); respondents with an average monthly income of NT$15,001–30,000 accounted for 41.28%; some respondents were unsure which industry their company belonged to, so “others” was the largest group (38.08%); most companies were private enterprises (95.02%); most of their positions were grassroots (52.67%); the type of job accounting for the largest proportion was administrative (21.35%).

**Table 2:** Sample demographics.

Measure	Item	Frequency (*n* = 281)	Percent (%)	Measure	Item	Frequency (*n* = 281)	Percent (%)
Gender	Male	94	33.45	Education	College/University	270	96.09
	Female	187	66.55		Graduates school	12	3.91
Marital status	Married	43	15.30	Nature of the company	Public sector	11	3.91
	Single	238	84.70		State-owned enterprise	3	1.07
Age	20 or below	38	13.52		Private sector	267	95.02
	21–25	100	35.59	Position	Part-time worker	66	23.49
	26–30	48	17.08		Grassroots	148	52.67
	31–35	41	14.59		Manager	62	22.06
	36–40	31	11.03		Senior management	5	1.78
	41–45	14	4.98	Monthly income (NTD)	0–15,000	38	13.52
	46 or above	9	3.20		15,001–30,000	116	41.28
Seniority (year)	1 or below	48	17.08		30,001–45,000	79	28.11
	1–5	136	48.40		45,001–60,000	37	13.17
	6–10	54	19.22		60,001–75,000	6	2.14
	11–15	23	8.19		75,001–90,000	3	1.07
	16–20	10	3.56		≥120,001	2	0.71
	21 or above	10	3.56	Nature of the job	R&D	7	2.49
Industry	Financial services industry	49	17.44		Quality control	4	1.42
	Traditional manufacturing	35	12.46		Customer service	38	13.52
	Information service industry	28	9.96		Manufacturing	12	4.27
	Medical services industry	5	1.78		Information	13	4.63
	High technology industry	24	8.54		Purchasing	6	2.14
	Food service	24	8.54		HR	7	2.49
	Real estate	9	3.20		Accounting/Cashier	35	12.46
	Others	107	38.08		Marketing/Sales	48	17.08
Company scale (number of people)	30 or below	103	36.65		Planning	5	1.78
	31–50	31	11.03		Administration	60	21.35
	51–100	39	13.88		Others	46	16.37
	101 or above	108	38.43				

### Reliability, Validity, and Model Fit

Each dimension and question was analyzed to evaluate the reliability, validity, and significance of estimated parameters for the observed variables. To establish the relationship between measurement indicators and potential variables, confirmatory factor analysis (CFA) tested the validity of the questionnaire (measurement indicators), and convergent validity and discriminant validity examined the reliability of each dimension. The results of reliability and validity analysis are shown in Table 3.

**Table 3:** Reliability and convergent validity.

Dimension	Cronbach’s *α*	CR	AVE
OJ	0.913	0.929	0.593
JS	0.833	0.900	0.749
OC	0.918	0.935	0.672
SN	0.788	0.863	0.612
PBC	0.924	0.938	0.655
SOCB	0.934	0.942	0.523

OJ, organizational justice; JS, job satisfaction; OC, organization commitment; SN, subjective norm; PBC, perceived behavior control; and SOCB, service-oriented organizational citizenship behavior.

Reliability refers to whether the measurement results reach consistency and stability, indicating the accuracy and reliability of the questionnaire. Consistency refers to whether the tests between internal questions are consistent with each other. Stability refers to whether the scores of the tests are consistent at different points in time. Reliability analysis uses Cronbach’s *α* and Composite Reliability (CR) to measure stability and consistency of a facet. Generally, the value of Cronbach’s *α* must be greater than 0.6, otherwise the scale must be redone. The overall Cronbach’s *α* value must be greater than 0.7 to have credibility, and a Cronbach’s *α* value of 0.8–0.9 is ideal, indicating a high degree of reliability (Nunnally, 1978). If the CR value of the potential variable is higher, the measurement variable is highly correlated, and the internal consistency is higher, which means that the item can effectively measure the potential variable, so the dimension has credibility. Fornell and Larcker (1981) recommends that a CR should be greater than 0.6, and it should be greater than 0.7 for a high degree of credibility (Hair et al., 1998). Table 3 shows that Cronbach’s *α* and CR for each dimension and item are higher than the recommended value, indicating good internal consistency.

Convergent validity refers to the degree of aggregation or correlation between multiple indicators (i.e., questions) that measure a single dimension. Cronbach’s *α*, average variances extracted (AVE), and CR were used as the basis for evaluating convergent validity (Hair et al., 1998). The AVE value is used to calculate the explanatory power of the potential variables to the variation of each measurement variable. Examine the reliability Higher AVEs indicate higher reliability and convergent validity. The AVEs are all higher than the recommended value of 0.7, so they have good convergence validity.

In addition, measurement of the items is based on relevant literature and theories, and questionnaires or measurement items used by experts and scholars are cited, and the content of this research is designed and modified into appropriate semantics. Therefore, this research questionnaire has considerable content validity.

Discriminatory validity confirms that the dimensions (potential variables) are indeed different. The Heterotrait-Monotrait ratio of correlations (HTMT) was adopted to test discriminant validity. Hair et al. (2021) proposed that HTMT values less than 0.90 indicate discriminant validity between dimensions. Table 4 shows that all values between two dimensions were less than 0.90. Thus, the dimensions in this study have discriminant validity.

**Table 4:** Heterotrait–Monotrait ratio of correlations (HTMT).

Dimension	OJ	JS	OC	SN	PBC	SOCB
OJ						
JS	0.706					
OC	0.746	0.617				
SN	0.484	0.338	0.552			
PBC	0.286	0.434	0.336	0.476		
SOCB	0.540	0.569	0.64	0.504	0.648	

OJ, organizational justice; JS, job satisfaction; OC, organization commitment; SN, subjective norm; PBC, perceived behavior control; and SOCB, service-oriented organizational citizenship behavior.

Furthermore, cross loadings can also be used to examine the discriminant validity to test the relationship between each item and different constructs. When items of one dimension are low related to other dimensions, there is discriminant validity between the dimensions. Conversely, in the cross-loading table, when the standardized factor loading of a question in a dimension should be more correlated with the other dimensions, it indicates discriminant validity (Hair et al., 2021). Results of the cross-loadings in this study met these criteria (see Table 5), indicating that the questions designed for this study had good discriminant validity.

**Table 5:** Cross loadings.

Dimension	Item	JS	OC	OJ	PBC	SN	SOCB
JS	JS 1	**0.870**	0.410	0.535	0.361	0.283	0.419
	JS 2	**0.878**	0.435	0.511	0.305	0.197	0.424
	JS 3	**0.849**	0.560	0.554	0.318	0.248	0.462
OC	OC 1	0.507	**0.866**	0.674	0.265	0.395	0.558
	OC 2	0.367	**0.695**	0.467	0.258	0.365	0.412
	OC 3	0.503	**0.825**	0.585	0.250	0.379	0.494
	OC 4	0.481	**0.836**	0.562	0.302	0.382	0.535
	OC 5	0.424	**0.786**	0.550	0.135	0.342	0.408
	OC 6	0.350	**0.823**	0.496	0.256	0.398	0.469
	OC 7	0.471	**0.895**	0.577	0.307	0.459	0.532
OJ	OJ 1	0.412	0.485	**0.680**	0.243	0.323	0.380
	OJ 2	0.489	0.554	**0.767**	0.233	0.310	0.390
	OJ 3	0.472	0.552	**0.735**	0.196	0.316	0.429
	OJ 4	0.528	0.602	**0.845**	0.167	0.333	0.412
	OJ 5	0.435	0.478	**0.766**	0.164	0.313	0.358
	OJ 6	0.509	0.482	**0.716**	0.295	0.289	0.363
	OJ 7	0.395	0.506	**0.748**	0.143	0.286	0.364
	OJ 8	0.482	0.504	**0.831**	0.141	0.296	0.325
	OJ 9	0.541	0.571	**0.826**	0.223	0.401	0.433
PBC	PBC 1	0.318	0.277	0.246	**0.727**	0.372	0.528
	PBC 2	0.345	0.287	0.212	**0.793**	0.334	0.484
	PBC 3	0.323	0.274	0.265	**0.864**	0.376	0.537
	PBC 4	0.293	0.272	0.196	**0.903**	0.371	0.492
	PBC 5	0.286	0.256	0.159	**0.870**	0.311	0.464
	PBC 6	0.273	0.168	0.173	**0.750**	0.252	0.446
	PBC 7	0.304	0.221	0.213	**0.757**	0.311	0.461
	PBC 8	0.318	0.249	0.216	**0.793**	0.282	0.478
SN	SN 1	0.272	0.449	0.369	0.186	**0.778**	0.361
	SN 2	0.245	0.390	0.337	0.302	**0.819**	0.309
	SN 3	0.117	0.301	0.268	0.344	**0.808**	0.315
	SN 4	0.224	0.325	0.309	0.438	**0.721**	0.376
SOCB	SOCB 1	0.421	0.787	0.587	0.244	0.356	**0.684**
	SOCB 2	0.404	0.724	0.588	0.276	0.389	**0.682**
	SOCB 3	0.434	0.626	0.497	0.472	0.371	**0.799**
	SOCB 4	0.429	0.691	0.572	0.330	0.417	**0.722**
	SOCB5	0.437	0.651	0.526	0.363	0.438	**0.765**
	SOCB6	0.416	0.445	0.411	0.475	0.381	**0.772**
	SOCB7	0.373	0.364	0.320	0.543	0.325	**0.756**
	SOCB8	0.326	0.283	0.281	0.549	0.283	**0.747**
	SOCB9	0.216	0.174	0.161	0.482	0.139	**0.592**
	SOCB10	0.337	0.233	0.166	0.518	0.224	**0.694**
	SOCB11	0.275	0.209	0.174	0.494	0.284	**0.716**
	SOCB12	0.360	0.289	0.265	0.504	0.304	**0.760**
	SOCB13	0.334	0.352	0.258	0.499	0.293	**0.794**
	SOCB14	0.356	0.304	0.265	0.452	0.282	**0.749**
	SOCB15	0.317	0.287	0.303	0.285	0.226	**0.574**

OJ, organizational justice; JS, job satisfaction; OC, organization commitment; SN, subjective norm; PBC, perceived behavior control; and SOCB, service-oriented organizational citizenship behavior. The bold values indicate the standardized factor loadings for each question.

When collecting questionnaires, an individual respondent may have cognitive similarity with the information, leading to common method variance (CMV). To avoid this problem, this study used anonymous surveys, concealed the meaning of questions, split up questions for each variable, and included reverse questions. Table 4 also shows that constructs of this study have construct validity, so the results are not significantly affected by CMV. Furthermore, this study uses the Harman’s One-Factor Test proposed by Podsakoff and Organ (1986) to test the severity of CMV. Explained variance of the marker variable was 34.105%, indicating that it was not related to the potential constructs. In conclusion, there was no severe CMV.

Final, as mentioned earlier, this study applies PLS-SEM to the analysis. Tenenhaus et al. (2004) proposed a global goodness of fit (GoF) for PLS-SEM to measure model fit. The equation is as follows, and the GoF is a value between 0 and 1. Wetzels et al. (2009) divided GoF into three levels, small (0.1), medium (0.25), and large (0.36).


$$GoF=\sqrt{\bar{AVE}\times \bar{R^{2}}}=\sqrt{0.642\times 0.330}=0.460$$

**Figure 2:**
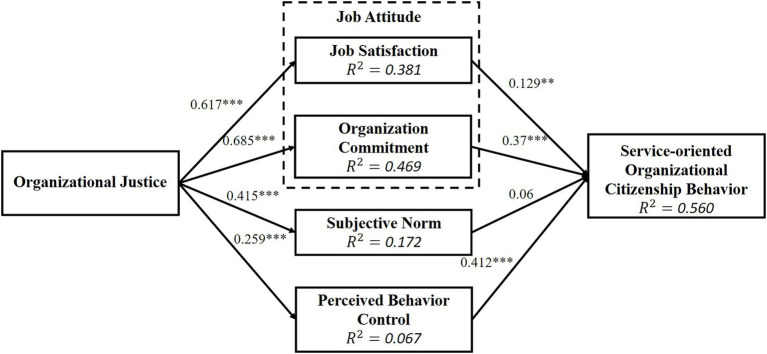
Test result of research hypothesis. **p < 0.01; ***p < 0.001.

This study put AVE (see Table 3) and *R*^2^ (see Figure 2) into this equation and obtained GoF = 0.460. This value is greater than 0.36, indicating that the model fits is significant and acceptable.

### Structural Equation Model Analysis

Structural equation model analysis (SEM) is based on the regression technique of multivariate technology, which conducts path analysis for potential variables to test fitness of the structural model. First, the items are reduced to fewer measurement indicators, the path is analyzed, the causal relationship between the variables is explored, and the various hypotheses are verified (MacCallum et al., 1994). Thus, SEM is based on a few measurement indicators to analyze various dimensions (potential variables) and evaluate causality.

Next, a model structure is established using SmartPLS for SEM analysis to explore relationships between the various dimensions. The result of path analysis is expressed by a path coefficient, which is divided into direct and indirect effects. Direct effect is the path coefficient from the independent variable to the dependent variable; indirect effect is the sum of products of the path coefficients from the independent variable to the dependent variable through the mediator. Effects of the overall potential variables are summarized in Table 6, and the results of the path analysis are shown in Figure 2. Except that *H7* has non-significant effect, the other hypotheses are supported (see Table 6). Moreover, the explanatory power (*R*^2^) is presented in Figure 2 (job satisfaction: *R*^2^ = 0.381; organizational commitment: *R*^2^ = 0.469; subjective norm: *R*^2^ = 0.172; perceived behavioral control: *R*^2^ = 0.067; service-oriented organizational citizenship behavior: *R*^2^ = 0.560), which is the percentage of the explained variance of the exogenous variables versus the endogenous variables.

**Table 6:** Direct effect.

Hypothesis		Path coefficient	CI	*t*-value	Result
H1	OJ → JS	0.617^***^	(0.509, 0.709)	12.046	Supported
H2	OJ → OC	0.685^***^	(0.609, 0.749)	18.749	Supported
H3	OJ → SN	0.415^***^	(0.297, 0.524)	7.225	Supported
H4	OJ → PBC	0.259^***^	(0.135, 0.374)	4.178	Supported
H5	JS → SOCB	0.129^**^	(0.031, 0.222)	2.604	Supported
H6	OC → SOCB	0.37^***^	(0.259, 0.478)	6.605	Supported
H7	SN → SOCB	0.06	(−0.032, 0.159)	1.219	Not supported
H8	PBC → SOCB	0.412^***^	(0.306, 0.506)	8.045	Supported

OJ, organizational justice; JS, job satisfaction; OC, organization commitment; SN, subjective norm; PBC, perceived behavior control; and SOCB, service-oriented organizational citizenship behavior.

* *p* < 0.05;

** *p* < 0.01;

*** *p* < 0.001.

Q-square is used to verify whether the model has predictive relevance. When Q-square is greater than 0, it indicates that the model has predictive relevance. In this study, Q-square values are all greater than 0 (job satisfaction: *Q*^2^ = 0.281; organizational commitment: *Q*^2^ = 0.311; subjective norm: *Q*^2^ = 0.100; perceived behavioral control: *Q*^2^ = 0.043; service-oriented organizational citizenship behavior: *Q*^2^ = 0.287), so the model has predictive relevance.

Finally, this study adopts bootstrapping, a method of resampling, to check the indirect effect according to the recommendation of Shrout and Bolger (2002). The indirect effect is significant if the value of *p* is less than 0.05 and the CI does not contain 0. Three mediation effect s are supported in this study (see Table 7), including (1) organizational justice → job satisfaction → service-oriented OCB; (2) organizational justice → organizational commitment → service-oriented OCB; and (3) organizational justice → perceived behavior control → service-oriented OCB. In short, job satisfaction, organizational commitment, and perceived behavior control have significant mediation effects.

**Table 7:** Indirect effect.

Dimension relationship	Path coefficient	CI	*t*-value	Result
OJ → JS → SOCB	0.08^**^	(0.02, 0.137)	2.663	Supported
OJ → OC → SOCB	0.254^***^	(0.177, 0.331)	6.376	Supported
OJ → PBC → SOCB	0.107^***^	(0.054, 0.158)	4.038	Supported

Number of bootstrap samples = 5,000. OJ, organizational justice; JS, job satisfaction; OC, organization commitment; SN, subjective norm; PBC, perceived behavior control; and SOCB, service-oriented organizational citizenship behavior.

* *p* < 0.05;

** *p* < 0.01;

*** *p* < 0.001.

## Conclusion

Based on the results of the research model above, this section further discusses the findings, describes management implications from the study findings, and suggests future research directions.

### Discussion

This study uses the conceptual framework of the DTPB to examine the impact of organizational justice on service-oriented employees OCB. Because this framework focuses on the discussion of behavioral intentions, the psychological impact of organizational justice must be reflected in behavioral performance. The model is discussed based on multi-dimensional factors, which are suitable for explaining and testing the purpose of this study.

The potential independent variables of organizational justice include procedural justice, interactional justice, and distributive justice. Employees want to be valued in their organization and expect good interpersonal relationships. If supervisors can respect and treat their subordinates fairly and peers can respect each other, employees will not have a feeling of unfairness, and have more retention commitment and work harder for the organization’s goal. In addition, if employees receive reasonable remuneration, they feel satisfied and willing to work hard. But if they feel they are exploited, they adjust self-efficacy and put less effort into their work. If employees think that they will get the same reward no matter whether they work hard or not, they will not work hard, thus lowering their self-worth commitment. If employees feel extreme unfairness and are unable to adjust, they may have diverging values or leave their jobs. Thus, the psychological factor of organizational justice is an important factor affecting behavior intention, and this study seeks to clarify the impact of organizational justice on service-oriented OCB. If an organization can better understand the perceptions of employees, it can formulate more appropriate management strategies.

This study first discusses the impact of organizational justice on perceived behavior control, subjective norms, and job attitudes (organizational commitment and job satisfaction). It then discusses the influence of perceived behavior control, subjective norms and job attitude on employees’ behavioral intentions. Service-oriented OCB is an extension of OCB and, as a common behavior in organizations, is the most appropriate way to explore current social situations. This study finds that organizational justice has a significant and positive effect on perceived behavior control, subjective norms, and job attitudes (organizational commitment and job satisfaction), indicating that employees attach great importance to organizational justice. In addition, the impacts of perceived behavior control, subjective norms, and job attitudes (organizational commitment and job satisfaction) on employee behavior were analyzed. Except for subjective norms, other factors have a significant and positive effect on behavioral intentions. This study argues that when organizations use cost-cutting measures (e.g., pay cuts, shorter working hours, unpaid leave), employees feel they are being treated unreasonably. When employees have the feeling of not being treated fairly, their work attitude (job satisfaction and organizational commitment) deteriorates and their confidence in doing their jobs decreases. Finally, these beliefs can impact their willingness to be proactive and enthusiastic in offering their services.

Job attitude includes organizational commitment and job satisfaction, which have important influences on attitude. Job satisfaction is a feeling of affirmation and being respected. Organizational commitment is the recognition that employees receive from the organization for their hard work. It influences the degree of effort and willingness of employees to stay in their jobs, and then forms the criteria for job attitude. The more affirmation employees receives from their organization, the more positive their job attitude will be. The subjective norms perceived by employees are not only influenced by ethics, but also by organizational norms. Because the behavior of employees should comply with organizational and ethical standards and must comply with the organization’s management system and rules.

Observing the findings in the previous section shows that job attitudes (organizational commitment and job satisfaction) have a significant and positive impact on service-oriented OCB. This means that employees with a good job attitude will be able to generate positive behaviors such as maintaining the organization’s image and providing enthusiastic service to customers. Furthermore, when employees are confident in their abilities to complete their work, they are more willing to take the initiative to provide good service to customers and interact with customers enthusiastically. When employees act as service staff and provide good service quality, customers will have a good impression of the organization and be willing to return, increasing profits of the organization. In addition, subjective norms are that employees comply with the norms of organization and ethics, and loyally provide consistent and standardized service quality to satisfy customers, so customers will be treated equally. Employees also play an intermediate role between the organization and customers and provide effective external publicity. Thus, employees with good subjective norms will also develop spontaneous OCB.

In service-oriented OCB, the main behavioral performance of employees is reflected in participation, service delivery and loyalty. For example, an employee’s sense of responsibility to the organization is a sign of loyalty. Proper service delivery means that employees will comply with organizational norms and provide consistent and standardized service quality to satisfy customers. Finally, participation means that employees provide excellent service that is spontaneous and goes beyond the norm. In addition, according to the theory of service profit chain, employee feedback increases the organization’s profits. But spontaneous feedback from employees is not easy, so an organization needs to recruit new members and increase training costs (Heskett et al., 1994). Hence, this study explores the influencing factors of service-oriented OCB by using DTPB. Empirical results show that perceived behavior control and job attitudes (organizational commitment and job satisfaction) affect service-oriented OCB. Nevertheless, these three factors are also affected by organizational justice. This study further verifies that organizational justice affects service-oriented OCB through perceived behavior control and job attitudes (organizational commitment and job satisfaction). Thus, these factors have mediation effects.

### Management Implications

This study examines this psychological perception of organizational justice to clarify the importance of organizational justice to organizations. For organizations to create profits, they must maintain their stability, and retaining good talent by reducing the turnover rate is important for organizational stability. Furthermore, a low staff turnover rate can reduce operation costs. Service-oriented OCB is the behavior of employees when they spontaneously work hard, beyond the scope of work norms. Employees are motivated to develop OCB, which is a continuous act of loyalty, and employees who perceive that the organization is treating them fairly are more likely to develop OCB. According to the theory of service profit chains, an organization’s profits can be increased by employee feedback. Managers hope that employees are willing to give back actively if the organization treats the employees fairly. When employees think that their hard work will be rewarded by corresponding compensation, they spontaneously give more. This creates a positive cycle, increasing the organization’s profits. This study confirms that when employees appreciate that their organization is treating them fairly, their job attitude and perceived behavior control are improved, which in turn affects service-oriented OCB. This will enable the organization to develop better. The study also confirms that when employees appreciate that they are treated fairly by the organization, it affects their perceived behavior control and job attitude, which in turn affects service-oriented OCB. As a result, organizations that treat their employees fairly are more likely to receive active feedback from employees that enable the organization to develop better. When the organization achieves its profit targets, its employees will also receive fair and reasonable compensation, forming a win-win situation for the organization and employees.

### Future Research and Suggestions

According to the research purpose, the respondents were employees in different industries, so this study could only understand some general perceptions of workers towards their organization. Consequently, this study proposes several future research directions. First, it is suggested that an overall assessment should be done for a single industry. Second, it is recommended that investigations be conducted at different points in time in the future, and there may be different findings. Third, more factors can be added to the evaluation, which may reveal more factors affecting the OCB to improve the organization-employee relationship, enhance the organization’s competitiveness, and create higher profits. Final, frontline service workers interact with customers. Zhu et al. (2021) studied the relationship between service satisfaction and customer citizenship behavior in e-tailing industry. Hence, this study suggests that this research framework can be used as a basis to discuss customer citizenship behavior in the future.

## Data Availability Statement

The raw data supporting the conclusions of this article will be made available by the authors, without undue reservation.

## Author Contributions

K-CT, Y-CL, and S-CC: conceptualization. TH and S-CC: methodology. T-HC, SK, and TH: validation; Y-CL and S-CC: formal analysis. Y-CL and S-CC: investigation. K-CT, T-HC, SK, TH, Y-CL, and S-CC: writing original draft preparation. TH, Y-CL, and S-CC: writing review and editing. Y-CL and S-CC: visualization. All authors contributed to the article and approved the submitted version.

## Conflict of Interest

The authors declare that the research was conducted in the absence of any commercial or financial relationships that could be construed as a potential conflict of interest.

## Publisher’s Note

All claims expressed in this article are solely those of the authors and do not necessarily represent those of their affiliated organizations, or those of the publisher, the editors and the reviewers. Any product that may be evaluated in this article, or claim that may be made by its manufacturer, is not guaranteed or endorsed by the publisher.
